# Latrine access and factors associated with it among people with physical disability in Kombolcha Town, Northeast Ethiopia: A mixed cross-sectional study

**DOI:** 10.1371/journal.pone.0270395

**Published:** 2022-06-24

**Authors:** Abuneh Getahun, Genet Gedamu Kassie, Tsion Samuel Bunare

**Affiliations:** 1 Kelela Woreda Health Office, South Wollo Zone, Kelela, Ethiopia; 2 Department of Environmental Health, School of Public Health, College of Medicine and Health Sciences, Bahir Dar University, Bahir Dar, Ethiopia; Prince Sattam Bin Abdulaziz University, College of Applied Medical Sciences, SAUDI ARABIA

## Abstract

**Background:**

Although all people have the right to access basic sanitation services, people with disabilities often face additional barriers to accessing the service compared to people without disabilities. Over the last few years, with the focus of the sustainable development goals on universal access to water supply, sanitation, and hygiene facilities, awareness of the need for programs to reach and benefit everyone has been growing. But the current level of access to latrines is not known.

**Objective:**

To determine latrine access and identify factors associated with it among people with physically disability in Kombolcha town, 2020.

**Methods:**

**A community-**based mixed cross-sectional study was conducted among physically disabled people in Kombolcha town in April 2020. Quantitative data was collected from 374 randomly selected study participants using structured interviewer-administered questioners. The key informant and in-depth interviews were conducted with purposely selected individuals.The bivariate and multivariable logistic regression analyses were conducted. Thematic analysis was used for qualitative data

**Results:**

Latrine access among people with a physical disability was found to be (22%), 95% CI (17.7–26.5). membershipto disability association (AOR = 2.162, 95% CI (1.231–3.799)),wealth status of study participants. (AOR = 4.169, 95% CI (1.96–8.864)) stigma and discrimination to get latrine in the last 12 months(AOR = 0.212, 95% CI (0.116–0.388)) and study participant’s knowledge to construct accessible latrine (AOR = 4.389, 95% CI (2.446–7.87)) werepredictor variables of latrine accessibility. Shared/publiclatrine, stigma and discrimination, poor wealth status,lack of own house, and lack of information provided regarding latrine accessibility supplementary barriersfor inaccessible latrinefrom In-depth and key informant interviews.

**Conclusion:**

Latrine access among people with a physical disability was found to be very low. Poor knowledge of accessible latrine construction, poor wealth status, stigma and discrimination, and not beinga member of a disability association increased the risk of latrine inaccessibility.

## Introduction

People with disabilities (PwDs) exist in every community in the world. Two-thirds of them live in low-income countries. Physical disabilities are one form of disability which mainly expressed as mobility and balance problems of individuals under specific impairments [1,2].

Globally, more than 1 billion people are estimated to have a disability, and among those, more than 110 million people with disabilities are not able to access improved WASH services. Persons with disabilities are known to have more difficulty accessing WASH services, and poorer countries have both restricted WASH access and greater disability prevalence [3].

An estimated 1.6 million people die from diarrheal diseases each year due to a lack of access to safe water and sanitation, and PwDs face additional barriers. All of the campaigns and initiatives to improve community-wide access to improved water and sanitation and to eliminate water, sanitation, and hygiene (WASH) associated diseases will not be successful unless PwDs are considered part of the general population [4].

Sanitation services and facilities are traditionally not user-friendly to PwDs. One such group is people with physical disabilities (PwPDs).The majority of PwDs do not need special facilities. Their needs can be met by ordinary services with a little extra thought.The additional cost of providing inclusive sanitation is found to be only 2 to 3% [5,6].

The prevalence of accessible latrines for PwPDs was found to be lower, Any type of latrine,even which is categorized under improved,might not be accessible for them due to the barrier in physical structure and design, environmental factors like distance to household, social and behavioral barriers like discrimination and stigma, and other socio demographical characteristics like age, sex, and income [7].

Ethiopia is one of the member states of the Sustainable Development Goal (SDG) signatories, which explicitly include disability and people s with disabilities, so it is imperative to promote disability inclusion to ensure access to water and sanitation for all, including people with disabilities, up to 2030. Some studies indicate that sanitation facilities designed for People with Physical Disabilitieswere found to be lower [8–10].

Almost all of water and sanitation surveys, including the Joint Monitoring Programme (JMP), do not cover the needs of disabled people. Similarly, in Ethiopia, bulky evidence is availableregarding the level of latrine access for the general population, but still, sufficient data wasn’t availableon the issue for those population groups living with physical disabilities. In parallel, possible interventional areas were not identified well.

This study was designed to be conducted in Kombolcha town because the town is one of the few highly industrialized towns in Ethiopia (more than 17 industries) and occupational hazards were higher (37%) for workers in those industries. This contributes to a higher prevalence of physical disability in the town [11,12]. Therefore, the results of this study can fill the existing gaps and determine the level of latrine accessibility and factors associated with it among people with physical disability in Kombolcha town through complementary quantitative and qualitative approaches.

## Methods and materials

### Study setting and period

A Community-based cross-sectional study supplemented by qualitative information was conducted in April 2020 in Kombolcha town which is one of the industrial towns located in Amhara regional state, north-east of Ethiopia, 378.5 kms to the North-East direction of Addis Ababa (capital of the country). Based on the 2007 national census, the estimated population of the study areas in 2019/20 is 156,138 of which 78,849 are females and 77,289 are males. The report from Kombolcha town labor and social affairs office shows there were 1224 people whose age was greater than 18 years and with some form of disability, out of the 748 of them were people with physical disabilities.

### Study design and population

A community-based cross-sectional study supplemented by qualitative information was employed, and all people living with a physical disability were the study population.

### Inclusion and exclusion criteria

All people living with physical disabilities who have lived at least six months in the town and whose age is greater or equal to eighteen years were included, Whereas those living with a physical disability that was severely ill during data collection were excluded.

### Operational definitions

#### People with physical disabilities

They are population groups for whatever reason cannot walk and may use a wheelchair, trolley, other mobility device or can walk with difficulty and need support from e.g. crutches, handrail, another person to lean on or can walk, but experience other physical weakness or lack of coordination, such as weak or erratic grip, or limited arm/hand movements [13].

**Improved latrine:**latrine facilities are those designed to hygienically separate excreta from human contact including pour flush latrine, ventilated improved pit latrine (VIP) andpit latrine with slab, otherwise ‘Un-improved sanitation type’ [14].

**Good latrine entrance:** wide enough and level enough (minimal or no difference between outside and inside) [15].

**Shared latrine:** A latrine which is used by two and more households in common [14].

**Latrine access to PwPDs:**the access to latrines which is at least improved type and permits the possibility to reach, enter, and use without any difficulty. Measured by Level and marked paths (≤ 6m) from the household, wide entrances to toilets (≥ = 1m), Enough space inside for a person, and her/his career to turn inside (≥ 1m^2^), Handrails, and grab bar [10,16].

**Knowledge of Accessible Latrine Construction**: This variable was measured using nine items. So, above the mean score reflects good knowledge, and below the mean score reflects poor knowledge [17].

### Sample size determination and sampling procedures

For quantitative data, the study sample size was determined using single population proportion formula (Z _α/2_)^2^p (1-p)/d^2^ with an assumption of 95% confidence interval, a margin of error (5%), and the proportion of PwPD with accessible latrine was found to be, p = 34% taken from the previous study done in Bahirdar, Ethiopia [8]. The final sample size considering a 10% non-response rate was 380. A simple random sampling technique was used to select participants from among the legally registered 748 PwPD under kombolcha town labor and social affairs office.For qualitative data, a total of 16 individuals,4 from people living with a disability for an in-depth interview, and 12 from sector offices working on WASH in the town) for key informant interviews were selected purposively.

### Data collection tools and procedures

The quantitative data was collected using **a** structured interviewer-administered questionnaire.The tool was comprised of socio-demographic characteristics, latrine access- related, and questions regarding the contributing factors to latrine access.Six diploma environmental health holders and two BSc environmental health holders (MSc holders) as data collectors and supervisors were recruited in the data collection process, respectively.

For the qualitative data, key informant and in-depth interview guides were adapted from different kinds of literature [4,18,19] and used for data collection. Two environmental health experts who had experience of qualitative data facilitation were recruited for data collection.Data was collected until no new information was raised and redundancies of ideas were recognized. All conversations during IDIs and KII were recorded and documented using an audio recorder and notebooks throughout the event with the consultation of the participants’.

### Study variables

The dependent variable was latrine access among people with physical disabilities (accessible or not accessible).The independent variables included socio-demographic variables (Age, sex, marital status, religion, education level, duration of living in the study area, and occupation), Social factors (stigma and discrimination to get and use latrine in the last 12 months), Institutional variables (accessible designs and government consideration and consultation during latrine designs) and Knowledge to construct accessible latrine.

### Data quality control

Quantitative data quality assurance was in place during questionnaire design, data collection, entry, and analysis.The questionnaire was prepared in English, then it was translated into Amharic (local language) and finally retranslated into English to check for consistency.The training was given to data collectors and supervisors on each data element.The questionnaire was pretested before the actual data collection with 15 PwPDs living in Dessie Town. The consistency and completeness of the data were checked on a daily basis by the supervisor and principal investigator. Thedata wereentered into Epi data version 3.1 to minimize errors during data entry.

Qualitative topic guides were originally prepared in English and then translated to Amharic and back to English to ensure the reliability of information obtained from KII and IDI. Key informants and in-depth interviews were conducted in the places in which participants were chosen for their freedom and increased confidence.

### Data analysis

Data from Epi-data-version 3.1 was exported into SPSS version 23.0 for analysis. Descriptive statistics like frequencies and percentages were calculated to see the overall distribution of the study subjects across the variables under the study. Bivariate logistic regression was conducted to assess the crude association and select important variables to be included in the final model. Finally, multivariable-binary logistic regressions were performed to identify factors associated with the access of latrines for PwPDs. The adjusted odds ratio (AOR) and their 95% CI are calculated to measure the association. A significance level of 0.05 was used to decide the significance of statistical tests.

Qualitative data was analyzed thematically.First, the raw qualitative data gained from the interview was transcribed from the audio version into text form. Next to this, the transcribed data was translated from Amharic into English. Then the translated data was coded and organized based on its dimensions and was analyzed and interpreted qualitatively.

## Ethical consideration

An ethical approval letter was obtained from the Ethical Review Board of Bahirdar University College of Medicine and Health Sciences. A permission letter was obtained from the disability association organization in kombolicha town. The respondents were informed about the purpose of the study, and their verbal consent was obtained. The respondents’ right to refuse or withdraw from participating in the study was fully maintained, and the information provided by each respondent was kept strictly confidential using codes.

## Results

### Socio-demographic andeconomic characteristics

A total of 380 study participants were included, with a response rate of 98.4%and 49.5% were members of the disability association. The mean age of the study participants was 33 (±11) years. The majority of 52.7% of the respondents were females, single 45.5% in marital status, Orthodox in religion 40%, a high school in the level of education 32%, and 54.8% in the poor wealth Status. The majority 38% were Students, 66.8% live ≤10 years in the study area and 88% has less than five family size (Table 1).

**Table 1:** Socio-demographic and economic characteristics of study participants in Kombolcha town in April 2020 (n = 374).

Characteristics	Frequency	Percent
Sex		
Male	177	47.3
Female	197	52.7
Family size		
< 5	329	88
≥ 5	45	12
Marital status		
Single	170	45.5
Married	161	43
widowed	35	9.4
Divorced	8	2. 1
Educational status		
Can’t read and write	106	28.3
Grade 1–8	85	22.7
Grade 9–12	119	31.8
Above grade 12	64	17.1
Wealth status		
Poor	145	38.8
Medium	125	33.4
Rich	104	27.8

## Prevalence of latrine access to PwPD

The prevalence of accessible latrines among PwPDs in the study area was found to be 22% (95% CI: (17.7–26.5). Among 374 study participants, 61.8% had any type of latrine, of which 43% were shared, 41.5% were unimproved types, 39%had more than six-meter paths from home, 50% had less than one-meter wide entrance, and 46% had less than one- meter square internal space,55% had no handrail, and 50% had no grab bar.

Among study participants living without an accessible latrine, 49% used open defecation. One hundred ninety-two (51.3%) of study subjects faced any form of stigma and discrimination forgetting a latrine in the last 12 months. The majority of participants (57%) didn’t get information regarding latrine accessibility in the last 12 months, 64.7% had not been consulted by the government during latrine design and construction, 59.4%had not been considered by the government t during latrine design and construction and 56.4% had poor knowledge about accessible latrine construction (Table 2).

**Table 2:** Latrine accessibility among PwPDs in Kombolcha town, April 2020.

Characteristics	frequency	Percent
Latrine accessibility (n = 374)		
no	291	78
yes	83	22
Latrine availability (n = 374)		
no	143	38.2
yes	231	61.8
latrine Owner (n = 231)		
private	123	53.2
Public/shared	108	46.8
Latrine distance from home (n = 231)		
≤6 meters	140	60.6
>6 meters	91	39.4
Entrance width(n = 231)		
<1 meter	115	49.8
≥1 meter	116	50.2
Space area of latrine(n = 231)		
< 1 meter squire	106	45.9
≥1meter squire	125	54.1
without contact with faces (n = 231)		
no	87	37.7
yes	144	62.3
Latrine have handrail?(n = 231)		
no	127	55
yes	104	45
Latrine have grab bar(n = 231)		
no	115	49.8
yes	116	50.2

### Factors associated with latrine accessibility for PwPDs

In binary logistic regression among sixteen variables study participant’s sex, age, wealth status, educational level, disability association membership, stigma and discrimination to get and use latrine in the last 12 months, latrine accessibility information in the last 12 month, the government consults during latrine design and construction,government consideration during latrine design andconstruction and knowledge of study participant to construct accessible latrine were selected as candidates for further multi-variable analysis.at the “p” value, less than 0.2.

In multivariable logistic regression disability association membership, stigma&discrimination to get and use latrine in the last 12 months,knowledge of study participants to construct accessible latrineand wealth status of study participants were significantly associated with the accessibility of latrine for PWPDs with ‘p’ value less than 0.05.

People living with physical disabilities who are in the rich wealth status were four times more likely to have an accessible latrine (AOR = 4.169, 95%CI (1.96–8.864)) than those who are in the poor wealth status. Similarly, people living with physical disabilities which are at the medium wealth status were 4 times more likely to have accessible latrine (AOR = 4.213, 95% CI (2.017–8.800)) than those which are at the poor wealth status. PwPDs who had a membership with disability association had 2 times more likely to have accessible latrine (AOR = 2.162, 95% CI (1.231–3.799)) than those who had nomembership. PwPDs did face some form of stigma and discrimination for getting or using a latrine in the last 12 months had a 79% greater risk of not having an accessible latrine (AOR = 0.212, 95%CI (0.116–0.388)) than their counterparts. The result was supported qualitatively by 10 out of 12 in-depth interviews, which showed thatthe stigma and discrimination for getting and using latrine is a common challenge ona day to day bases and it was higher when they try to get the latrine in public areas and institutions. The main forms of stigma and discrimination were lack of interest to use the latrine after physically disabled people used embarrassment to get the latrine and locking the latrine. PwPDs who had good knowledge of constructing accessible latrines were 4 times more likely to have accessible latrines (AOR = 4.389, 95% CI (2.446–7.87))than their counterparts (Table 3).

**Table 3:** Bi-variable and multi-variable logistic regression on factors associated with latrine accessibility among PwPDs in Kombolcha town in April 2020 (n = 374).

characteristics	Category	latrine accessibility		Odds ratio (OR),95%	
		Accessible (%)	Inaccessible (%)	Crude (‘*P*’ = 0.2)	Adjusted(‘*P’* = 0.05)
sex	Male	47(12.6)	130(34.8)	1.617[0.989–2.644]	1.336[0.755–2.366]
	Female	36(9.6)	161(48)	1	1
Age	18–30	26(7)	113(30.2)	0.5[0.257–0.998]	0.468[0.205–1.066]
	31–43	27(7.2)	104(27.8)	0.57[0.29–1.124]	0.485[0.210–1.123]
	44–56	10(2.7)	30(8)	0.733[0.301–1.785]	0.548[0.185–1.625]
	≥ 57	20(5.3)	44(11.8)	1	1
Wealth status	poor	14(3.7)	131(35)	1	1
	medium	37(9.9)	88(23.5)	3.934[2.010–7.702]	4.213[2.017–8.800]
	Rich	32(8.6)	72(19.3)	4.159[2.084–8.297]	***4*.*169[1*.*96–8*.*864]***
educational level	Cannot read and write	18(4.8)	88(23.5)	1	1
	1–8	18(4.8)	67(18)	1.313[0.635–2.716]	0.990[0.420–2.333]
	9–12	31(8.3)	88(23.5)	1.722[0.898–3.304]	1.644[0.759–3.559]
	Certificate and above	16(4.3)	48(12.8)	1.63[0.762–3.484]	1.156[0.464–2.880]
disability association membership	no	36(9.6)	181(48.4)	1	1
	yes	47(12.6)	110(29.4)	2.148[1.313–3.523]	***2*.*162[1*.*231–3*.*799]***
stigma &discriminations	no	62(16.6)	120(32)	1	***1***
	yes	21(5.6)	171(45.7)	0.238[0.138–0.411]	***0*.*212[0*.*116–0*.*388]***
latrine accessibility information	no	42(11.2)	171(45.7)	1	1
	yes	41(11)	120(32.1)	1.391[0.853–2.27]	1.239[0.697–2.205]
gov’t consult in latrine design	no	49(13)	193(51.6)	1	1
	yes	34(9)	98(26.2)	1.367[0.828–2.254]	1.487[0.831–2.661]
gov’t consider insanitation program	no	39(10.4)	183(48.9)	1	1
	yes	44(11.8)	108(28.9)	1.912[1.168–3.128]	1.396[0.785–2.480]
latrine construction k/ge	poor	23(6)	188(50.3)	1	**1**
	good	60(16)	103(27.5)	4.762[2.782–8.149]	***4*.*389[2*.*446–7*.*87]***

### Qualitative results

#### Summary of In-depth interviews

A total of four [4] themes were identified from in-depth-interview data to explore the barriers to latrine accessibility among PwPDs qualitatively.

**Theme 1: Stigma and Discrimination:**Out of total in-depth interviewees,10 of them had similar ideas regarding stigma and discrimination to get latrine. The stigma and discrimination in the community start from their own families.

A 22- year -old woman said that *“I am moving with a wheelchair and living with my uncle’s family*. *The latrine has higher steps which inhibits me from entering with my wheelchair*.*it is also difficult to get the latrine at day time because they always ordered me to go to the latrine only at night after all family members used”*.

**Theme 2:Wealth Status;** ten out of twelve in-depth interview participants show that they were living with lower hand to mouth daily income sub standardly. They hadn’t had extra money to save for such needs. Even sometimes, some of them might miss their normal lunch or dinner.

A 26- year-old woman said, “*If I am rich*, *I will modify my latrine first*.*”*

**Theme 3: Shared/Public Latrines:** All the interviewees agreed that public/shared latrines were challenges for them related to lack of freedom and cleanliness.

A 22-year- old girl high school student said, “*I used a public latrine with our neighbors*. *The latrine is very dirty*, *especially in the afternoon and at night*. *I had no freedom to use it during the day*.*my hands*, *legs and cloths had contact with dirty matter many times”*.

**Theme 4: Lack Of Own House;** fifty percent of interviewees in an in-depth interview shows they wereliving in a small rented house. They couldn’t make any adjustments to their living environments, including their living class. Even they couldn’t find a rental house with such infrastructure purposely designed to include them.

A 38- year- old man who moves with the help of his knee and arm said “*I have lived here in this town for more than 10 years*. *And I was changed my rental house more than six times*.*it was impossible to find the latrine accessible to me*. *I usually defecate in ditches and sometimes open fields*. *Now I chose to rent at the end of the town to get free space easily*.*”*

#### Summaryof key informant interview

A total of three themes were identified in the key informant interviews.

**Theme 1: Wealth Status;**all the KI interviewees had similar views that PwPDs are poor people with few exceptions.

According to the town municipality office sanitation focal and health office sanitation officer,“*we know they are among the poorest people in the town*, *who need special treatment in all aspect including their latrine access*.

**Theme 2: Stigma And Discriminations**:All KII participants were assured that PwPDs have to face different types of stigma and discrimination to get latrine access.

The heads of town disability association display that “*stigma and decimation of PwPDs to get a latrine were common and the problem is higher in households which have communal/shred latrine”*.

Theme 3: Lack of information provision regarding accessible latrines;

All key informant interview participants agreed that technical support and information provision about accessible latrine designs to PwPDs were null and not evaluated well.

But the town health office sanitation officer said that *“no one in government including our office*, *has told them how they can modify and make their latrine accessible*.

## Discussions

This study revealed that only 22% of participants had an accessible latrine. This prevalence is consistent with a study conducted in India (26%) and Malawi (24%) [7]. It was lower than the United Nations development report on developing nations, which states 20% of people with disabilities had not accessible latrine and out of 45000 latrines, 31% were not accessible to wheelchair users [20]. This difference in results might be due to the differences in latrine access measuring indicators. It is also somewhat lower than the results of previous similar studies conducted in Ethiopia, Gondar (29%), and Bahirdar (34%) [8,10] and it might be due to differences in socio-demographic characteristics like educational status occupation, and income levels. Whereas it is higher than the results of another Indian study, only (10%) of PWPDs had accesse to latrines [1]. This deviation in results might be because of differences in socio-demographic characteristics of PwPDs.

The SDG was ratified to practice the“leave no one behind” principle for all developmental goals.Specifically, SDG 6 reflects universal WASH access. Vulnerable population groups like people with physical disabilities were stated to gain special treatments [9]. However, results of this study indicate that people with physical disabilities are under multiple challenges to meet their latrine needs, particularly the need of short distance of latrine from home, wide latrine entrance and a spacious enough latrine, use of latrine without contact with dirt and faces and building their ownprivate latrines and latrines with handrails and grab bars. This lower prevalence of accessible latrines to PwPDs might be due to most of the existing latrines were traditional type, some were at a longer distance from home, with rough paths, narrow entrances,narrow space inside, steps to latrines, slippery surface, and an absence of grab bars and handrails [21]. Another possible explanation might be that physically disabled people are poor with a high unemployment rate, so they cannot afford basic services including their sanitation needs [20,22].

PwPDs who are at the poor wealth status had a higher risk of having inaccessible latrines than their rich wealth status counterparts.The finding was supported qualitatively by showing that in-depth interview participants outlined their poorness inhibited them from not to modifing and making latrine accessible.The result was also supported by the study conducted in Nepal that the poorest disabled people experience higher challenges in sanitation than those disabled people with good economic status and the review conducted on low and middle-income countries, which shows PwDs in the poorest status had more chances than the wealthiest status to practice open defecation [4,23,24].The possible rationale behind this might be that poor PwPDs do not have enough money to pay for personal costs and the resources needed to construct an improved accessible latrine [24]. According to Basic Need Approach (BNA), an absolute measurement of poverty, basic needs are not only the traditional (food, cloth. and shelter) but also sanitation and education and health. Poor people are those whose income is below the poverty line, (fulfilling the above basic needs) [25]. It might also due to the reason that poor people did not have their own house and can not construct and modify the latrines as they wanted without the interest of the renter [26].The lack of own house was also identified by the qualitative part of the current study as the main constraints on PwPDs to having an accessible latrine.

PwPDs who were not members of with disability association had more risk of being inaccessible to latrines than those who had a membership. The summary of both in-depth and key informant interviews in the current study has similar reflections to this result. This result is alsosupported bythe study conducted in Gondar, Ethiopia that shows PwPDs who had a membership with disability association more likely to have accessible latrine than those who had not disability association membership [10]. It might be due to disability associations struggles on the right of members including in latrine accessibilities. The other possible explanation can be PwPDs who had a membership to the association had an opportunity to gain information regarding latrine accessibility [27].

PwPDs who didn’t face some form of stigma and discrimination for getting latrine in the last 12 months had lower risk of having an inaccessible latrine than those who faced inaccessible latrines.This result was supported by qualitative parts of this study and findings of other reviews conducted on low and middle-income countries that showed people with physical and other disabilities may tend to take longer time to use the latrine and stigmatizing experiences especially in communal latrines. This pushes them to practice the stigma associated with open defecation [4,28].The possible explanation might be due to people who are discriminated and excluded had less chance to decide on their needs [26] And hence they cannot modify or arrange the physical environments of latrine as they want. It might be also due to the reason that mostly discriminated people are hidden inside the home by their families to keep the name and position of family and they are dependent on others so that impossible to influence their demand. Everything could be done by the volition of others [29].

PwPDs who had poor knowledge of how to construct accessible latrine were more likely to have inaccessible latrines than those who had good knowledge. In the key in-depth interview of this study, participants outlined that a lack of information provision on accessible latrine designs was identified as one of the main barriers to having and getting accessible latrine. Even though limitation of both qualitative and quantitive reports to compare this finding, but this might be due to those PwPDs who had active involvement in various issues the community had exposure and chance of gaining multiple skills needs for their day to day bases [26].

## Conclusion

In this study, latrine access among people living with physical disabilities was found to be very low. The disability association membership, wealth status of study participants, stigma and discriminations to get and using latrines in the last 12 months, and knowledge to construct accessible latrine were predictors of latrine accessibility for PwPDs.Stigma and discrimination to get latrine, poor wealth status of PwPDs, shared/public latrine, lack of own houseand lack of information provided regarding latrine accessibility were a supplementary finding for quantitative results and identified as a barrier to latrine accessibility from qualitative findings.

## Supporting information

S1 Survey Survey tool. (DOCX)

S1 Dataset Minimal data set. (SAV)

## Abbreviations

AOR: Adjusted Odds Ratio
CI: Confidence Interval
COR: Crude Odds Ratio
JMP: Joint Monitoring Programme
IDIs: Indepth interviews
KII: Key Informant Interviwes
PwPDs: People with Physical Disabilities
SDG: Sustainable Development Goal
WASH: Water, Sanitation and Hygiene
WHO: World Health Organization
