# Supplementary material for: Latrine access and factors associated with it among people with physical disability in Kombolcha Town, Northeast Ethiopia: A mixed cross-sectional study
Source: PLoS One. 2022 Jun 24;17(6):e0270395. doi: 10.1371/journal.pone.0270395 (PMC9231769; doi:10.1371/journal.pone.0270395)
Supplement: S1 Survey — (DOCX) [file pone.0270395.s001.docx]

## English version participant questionnaire

Questionnaire code_______________

Date of data collection___________________

| **Part one: socio demographic characteristics** | | |  |
| --- | --- | --- | --- |
| S.no | Questions | answer |  |
| 101 | What is the Sex? | 1 Male  2 Female |  |
| 102 | What is your age in year? | ________ |  |
| 103 | What is your religion? | 1 Orthodox  2 Muslim  3 Protestant  4 Other (specify |  |
| 104 | How many member of household are there in the home? | -------- |  |
| 105 | How long do you live in this town in year? | ------------- |  |
| 106 | What is your current marital status? | 1 Single  2 Married  3 Divorced  4 Widowed |  |
| 107 | Occupation/job of study participants | 1 Government  employ  2 Merchant  3 Farmer  4 Student  5 Private employ  6 House wife  7 Daily labor  8 Other specify |  |
| 108 | Educational status | 1 Cannot read and write  2 Primary(1-8)  3 Secondary(9-12)  4 Certificate and above |  |
| 109 | Are you the member of disability association? | 1= no 2= yes |  |
|  |  |  |  |

**PART 2**: **Wealth index assessment Questionnaire**

| S.no | Questions | Response |
| --- | --- | --- |
| 201 | Where do you live? | 1. Own house 2. Rented house |
| 202 | Number of rooms in the dwelling place | ________________ |
| 203 | What is the wall of the house made off? (check by observation) | 1. Wood but not have mud 2. Wood with mud 3. Mud only 4. Wood and cement 5. Blocket 6. Others specify__________ |
| 204 | Observe that which material the house roof is made off? | 1. Grass/ leaf 2. corrugated iron |
| 205 | What is the floor of the house made off?(check by observation) | 1. Natural ground 2. Muck/smooth by cow’s faces 3. Wood 4. Cement 5. Other Specify)_____________ |
| 206 | What is your main source of cooking fuel? | 1. Firewood /Animal dung 2. Charcoal 3. Electricity 4. Kerosene/gas 5. Others Specify___________ |
| 207 | What is the main sources of lighting? | 1. Kerosene 2. Electricity 3. Solar 4. Candle 5. Others specify_____________ |
| 208 | Radio | 1 No  2 yes |
| 209 | Television | 1 No  2 yes |
| 210 | Fridge | 1 No  2 yes |
| 211 | Chair | 1 No  2 yes |
| 212 | Table | 1 No  2 yes |
| 213 | Bed and mattress which made from cotton spring | 1 No  2 yes |
| 214 | Mobile | 1 No  2 yes |
| 215 | Cycle | 1 No  2 yes |
| 216 | Motor cycle | 1 No  2 yes |
| 217 | Horse’s cart | 1 No  2 yes |
| 218 | Bajaj/car | Yes  No |
| 219 | Bank book | Yes  No |
| 220 | Sofa | Yes  No |

**Part 3 latrine accessibility related questions**

| 301 | Does the household have latrine? | 1=no  2=yes |
| --- | --- | --- |
| 302 | If” yes’ for Q No 301 who is the owner of it? | 1= private  2=public/shared |
| 303 | If “yes” for Q No 301 What type of latrine is it? | Flush/Pour flush  1 = Flush to piped sewer  2 = Flush to septic tank  3 = Flush to pit (latrine)  4 = Flush to somewhere else  Pit latrine  5=ventilated improved  6 = Pit latrine without slab  7 = Composting latrine  8 = Bucket latrine  9 = Hanging latrine  88 = Other (specify)----------------- |
| 304 | If “yes” for Q No 301 do you use the same latrine as other member of your household? | 1=no  2=yes |
| 305 | If” no” to question 304, where do you use latrine? | 1= pour flush  2= improved pit  3=unimproved pit  4=0pen field/bush |
| 306 | If” no” to question 304, why do you use a different facility from other members of your household? | 1 = It would be physically difficult/impossible for me  2 = Other people would not like it/I am not allowed  3 = I would be embarrassed  /people would laugh at me  4 = People would abuse me verbally or physically  5 = It's too far  6 = The path is too difficult  7 = I can't get into the toilet e.g. the entrance too narrow/step too high  8 = I have difficulty squatting and there is nothing to hold onto  9 = I crawl on hands and knees and the ground is too dirty  10 = Lack of privacy  11 = I feel unsafe  88 = Other (specify) |
| 307 | How far does the latrine from house | 1 = equal or < 6meters  2 = >6 meters |
| 308 | Do you use any of the following assistive devices during your movement to latrine | 1 Wheelchairs  2 Crutchers  3 Artificial limb  4 Someone’s assistance  5 No, but I need |
| 309 | How wide is the entrance of the latrine? | 1= less than 1 meter  2= equal or more than 1 meter |
| 310 | How much spacious the latrine room? | 1= <1m^2^  2= equal or > 1m^2^ |
| 311 | Does the latrine enable you to use it without assistance from other person? | 1=no  2=yes |
| 312 | Does the latrine facility enable you to use it without coming into contact with faeces or urine? | 1=no  2=yes |
| 313 | Does the latrine has handrail? | 1=no  2=yes |
| 314 | Does the latrine has grab bar? | 1=no  2=yes |
| 315 | Does the latrine accessible for PWPDs? | 1 no  2 yes |
| 316 | Are you facing any stigma and discrimination in access of latrine? | 1=no  2=yes |
| 317 | Did you get information about accessible latrine to you In the past 12 months | 1=no  2=yes |
| 318 | Did you consulted and participated by the government during latrine design | 1=no  2=yes |
| 319 | Does the government considered your latrine desire and need? | 1=no  2=yes |

**Part 4 Accessible Latrine construction knowledge related questions**

| 401 | Have you ever heard about the construction of accessible latrine designs for PWPDS? | 1=no 2=yes |
| --- | --- | --- |
| 402 | If yes for Question yes 401 From where did you hear about it? | 1. Mass media  2.Health workers  3.NGOs workers  4.Relatives/family  5.Friends  6.diaablity associations  88. Other (specify |
| 403 | Do you know the construction of accessible latrine designs for PWPDS? | 0=no 1=yes |
| 404 | Which latrine modifications did you know to make latrine accessible for PWPDS? | 1.road to latrine  2. steps  3.entrance width  4. grabs  5..handrails  6.internal space  88. Other (specify |
| 405 | Can shared latrines accessible for PWPDS? | 1=no 2=yes |
| 406 | The distance from house to latrine should not be more than 6meters for PWPDS. | 1=no 2=yes |
| 407 | The handrails and grapes should be installed to latrines for PWPDS. | 1=no 2=yes |
| 408 | The entrance of the latrine should not be less than one meter wide for PWPDS. | 1=no 2=yes |
| 409 | The minimum internal space of the latrine for PWPDS should not be less than one squire meter | 1=no 2 yes |
| 410 | Latrine for PWPDs needs somewhat little amendments than general population | 1=no 2=yes |
